# 

**Published:** 1851-08

**Authors:** 


### CONTENTS

### OF NO. II.

### ORIGINAL COMMUNICATIONS.

### ESSAYS AND CASES.

Art. I.—Spontaneous Combustion. Translated from the Archives Generales de Medicine. By P. K. - - 93

Art. II.—Remarks on a peculiar appearance of the Tongue in Malarial Disease; read before the Greensboro' Medical Society. By Dr. Thos. C. Osborne, of Erie, Ala. 109

Art. III.—A brief notice of the Treatment of Dysentery; the Honey Bee as a Diuretic; and Warm Water in Colic. By Dr. M. H. Fee, of Heltonville, Indiana. - - 123

### REVIEWS.

Art. IV.—On Diseases of Menstruation and Ovarian Inflammation, in connexion with Sterility, Pelvic Tumors, and Affections of the Womb. By Edward John Tilt, M.D., Physician to the Farrington General Dispensary, and to the Paddington Free Dispensary for the Diseases of Women and Children. "Omne animal ab ovo." 126

Art. V.—Operative Surgery. By Frederic C. Skey, F.R.S. 142

Art. VI.—The Dissector; or, Practical Surgical Anatomy. By Erasmus Wilson, author of a "System of Human Anatomy." With 115 illustrations. Edited by Paul B. Goddard, M.D. A new and improved edition. - 148

Art. VII.—A Treatise on Dislocations and Fractures of the Joints. By Sir Astley Cooper, Bart., F.R.S. A new edition, much enlarged. Edited by Bransby B. Cooper, F.R.S., Surgeon to Guy's Hospital. With additional observations, and a memoir of the author. - - 148

Art VIII.—On the Theory and Practice of Midwifery. By Fleetwood Churchill, M. D., M. R. I. A., etc. With notes and additions, by D. Francis Condie, M.D., Secretary of the College of Physicians, etc., etc. - 150
Art. IX.—Urinary Deposits; their Diagnosis, Pathology, and Therapeutical Indications. By Golding Bird, A.M., M.D., F.R.S., F.L.S., etc., etc. Second American, from the third revised and enlarged London edition. 151

SELECTIONS FROM FOREIGN AND HOME JOURNALS.

On the Purity and Use of Chloroform. - - - - 153
Charge against a Medical Practitioner. - - - - - 165
Suit for Malpractice. - - - - - - - 168
On a substitute for McMunn's Elixir of Opium. - - 170
Complete absence of the Uterus. - - - - - 171
Incontinence of Urine. - - - - - - 172
Case of Poisoning by Laudanum. - - - - - 172
Artificial Vichy Water. - - - - - - 173
Measures for Combatting Sterility. - - - - 173

ORIGINAL INTELLIGENCE.

The Southern Medical and Surgical Journal. - - - 174
Sanitary Reform. - - - - - - - - 178
Health of Louisville. - - - - - - - 181
Gross on the Urinary Organs. - - - - - 181
Bad Midwifery. - - - - - - - - 182
The Retraction. - - - - - - - - 182
Kentucky State Medical Society. - - - - - 182
Circular to the Physicians of Tennessee and Kentucky. - 183
University of Louisville. - - - - - - 184
To Correspondents. - - - - - - - - 184

